# Effect of Photobiomodulation on Salivary Cytokines in Head and Neck Cancer Patients with Oral Mucositis: A Systematic Review

**DOI:** 10.3390/jcm13102822

**Published:** 2024-05-10

**Authors:** Marwa Khalil, Omar Hamadah, Maher Saifo, Hasan Khalil, Mowaffak Adi, Faris Alabeedi, Omar Kujan

**Affiliations:** 1 Department of Oral Medicine, Faculty of Dental Medicine, Damascus University, Damascus P.O. Box 30621, Syria; marwa.khalil@damascusuniversity.edu.sy (M.K.); omar.hamadah@damascusuniversity.edu.sy (O.H.); 2 The Higher Institute for Laser Research and Applications, Damascus University, Damascus P.O. Box 30621, Syria; 3 Faculty of Medicine, Medical Oncology, Damascus University, Damascus P.O. Box 30621, Syria; maher.saifo@damascusuniversity.edu.sy; 4 Albairouni University Hospital, Damascus University, Damascus P.O. Box 30621, Syria; 5 Department of Microbiology and Biochemistry, Tishreen University, Lattakia P.O. Box 2230, Syria; hasan.khalil@tishreen.edu.sy; 6 Shining Horizons Dental Center, Inaya Medical Colleges, Riyadh 13541, Saudi Arabia; drmuafak.shdc@inaya.edu.sa; 7 UWA Dental School, The University of Western Australia, Nedlands, WA 6009, Australia; faris.alabeedi@research.uwa.edu.au

**Keywords:** photobiomodulation, low-level laser, salivary cytokines, oral mucositis

## Abstract

**Background:** Oral mucositis is a common and distressing side effect of head and neck oncology treatment. Photobiomodulation therapy can be utilized to prevent and treat oral mucositis. Its impact on salivary cytokines has yet to be thoroughly investigated. This is the first systematic review aiming to evaluate the effect of photobiomodulation on salivary cytokines in patients undergoing anticancer treatment. **Methods:** Numerous data resources, from the Web of Science, Embase, ScienceDirect, PubMed, Cochrane Library, and Scopus were sought. Articles published up until February 2024 were included if they met the following inclusion criteria: clinical trials reporting the effect on salivary cytokines in patients undergoing anticancer therapy. The methodological quality was assessed using several appraisal tools. **Results:** Four studies were deemed eligible for inclusion. All the studies were conducted in Brazil and used an InGaAlP diode laser with a wavelength of 660 nm. The included studies had a relatively low risk of bias. The head and neck cancer patients’ salivary cytokines that were assessed by the studies, along with photobiomodulation therapy, included IL-12p70, TNF-α, IL-6, IL-8, IL-10, CXCL8, and IL-1β. The results varied among the studies. **Conclusions:** Our results show that photobiomodulation demonstrated positive results for reducing the severity of OM in all the included studies. Among the examined salivary cytokines, IL-6 is the most relevant cytokine for oral mucositis development and severity. A variation in the cytokine levels between the studies was noted due to differences in the type of anticancer treatment and saliva sampling.

## 1. Introduction

Oral mucositis [OM] is a highly distressing and common side effect of the non-surgical treatment of malignancies. It may result from systemic chemotherapy, radiation therapy, a combination of both, or in patients who undergo hematopoietic stem cell transplantation. OM is observed in almost 30–40% of head and neck cancer patients who undergo chemotherapy alone [1,2]. Whereas, patients receiving hematopoietic stem cell transplantation exhibit an increased occurrence percentage of 60–85%, and almost 90% of patients who undergo radiotherapy and chemotherapy together [3,4].

Oral mucositis develops via direct and indirect pathogenetic processes [5]. In the early stages, direct DNA damage occurs due to the breakage of DNA strands, thus causing epithelial basal cell death and the accumulation of reactive oxygen species, leading to complex bioreaction events and subsequent mucosal damage. Reaction oxygen species [ROS] mediate the activation of NFκB and the release of pro-inflammatory cytokines, such as IL-6 and TNF-α, which, in turn, stimulates pathways that destroy surrounding epithelial cells and fibroblasts [6]. Subsequently, tissue damage and programmed cell death occur. Deep and painful ulcerations extend from the epithelium to the submucosa, with nerve endings exposed and rapidly colonized by oral bacteria and latent or secondary viral infections that effectively contribute to mucositis because they stimulate the secretion of more pro-inflammatory cytokines [6].

Several studies have analyzed salivary cytokines in patients who have developed oral mucositis. A systematic review by Diesch et al. concluded that TNF-α, IL-2, IL-6, and IL-1β pro-inflammatory cytokines are interrelated with the severity of and damage to oral mucosal tissue; this is of great practical importance for the early detection of mucositis without needing an in-depth oral examination. An oral examination is often challenging, particularly when patients have limited mouth-opening ability or are in significant pain. Examining the salivary cytokines can enhance patients’ quality of life by knowing when to interfere and administering early therapeutic interventions [7].

Photobiomodulation (PBM) therapy is the application of light in the wavelength range of [600 nm–1000 nm] to injured or potentially injured tissue for pain relief, inflammation reduction, and improvement of the healing process [8,9]. The World Association of Photobiomodulation Therapy [WALT] group has demonstrated PBM’s therapeutic and prophylactic prospects for cancer therapy side effects [10]. The International Society of Oral Oncology and The Multinational Society for Supportive Care in Cancer (MASCC/ISOO) group have also recommended using PBM to manage oral mucositis [11,12].

Several systematic reviews have confirmed photobiomodulation therapy’s effectiveness for preventing and treating oral mucositis [13,14,15,16]. However, the effects of PBM at the cellular level and the biochemical response, especially of the cytokines, still need to be fully understood. This may impact the delivery of this therapeutic approach in future studies. Therefore, this is the first systematic review aiming to evaluate the effect of photobiomodulation on salivary cytokines in patients undergoing anticancer treatment.

## 2. Methods

This systematic review was prepared according to the Preferred Reporting Items for Systematic Reviews and Meta-Analyses—PRISMA guidelines [17], and has a registration number (CRD42023441214) with the International Prospective Register of Systematic Reviews (PROSPERO).

### 2.1. Study Design

This review summarizes all human trials on the effect of photobiomodulation on salivary cytokines in cancer patients undergoing radiotherapy alone or in combination with chemotherapy. 

The PICO framework was utilized to formulate the research question. It is based on the following: Participants—cancer patients who underwent radiotherapy alone or in combination with chemotherapy who developed OM. Intervention—photobiomodulation therapy. Comparison—placebo, nothing, or other preventive or therapeutic measures. Outcome—cytokine levels in saliva. 

### 2.2. Search Strategy

All papers relevant to this topic were searched for using MeSH terms and related free terms, and were identified in the following databases: Medline via PubMed, Scopus, ScienceDirect, and Cochrane Library. No lower date limit was set, and an upper date limit of February 2024 was established. There was no language restriction imposed. The keywords used in this systematic review were photobiomodulation, low-power laser, low-level laser therapy, low-level light therapy oral mucositis, oral stomatitis, chemotherapy-induced oral mucositis, chemotherapy-induced oral stomatitis, cytokine, IL, interleukin, cytokines, saliva, and salivary. The Boolean term used for the search process was “AND, OR”, which was used to ensure comprehensive results. After a rigorous analysis, only four articles met the criteria and were deemed eligible. This thorough approach ensures the reliability and validity of the clinical results by ensuring that the final evaluation is based on the accuracy and relevancy of the studies.

### 2.3. Inclusion Criteria

Published clinical trials showing the effect of photobiomodulation on salivary cytokines in patients undergoing anticancer therapy were deemed eligible for inclusion. Thus, this includes chemotherapy and radiotherapy.

### 2.4. Exclusion Criteria

Published articles were excluded, including systematic reviews, meta-analyses, observational studies, case reports, case series, animal research, in vitro studies, study protocol clinical trials, cost-effectiveness randomized clinical trials, editorials, opinions, and conference abstracts.

### 2.5. Study Selection

Three authors (M.K., O.H., and F.A) independently performed the study selection. The titles and abstracts of all the papers were first meticulously reviewed and assessed, followed by an independent selection process by the authors for the studies that met the inclusion criteria, and then an evaluation process for all the articles. The full text of the article was the primary determinant in the final selection process. A PRISMA flowchart was generated for the studies included in this systematic review [18].

### 2.6. Data Extraction

The following data were extracted from the selected studies: author, publication date, type of tumor, anticancer therapy, sample size, comparison group, kind of PBM device, OM evaluation methods, type of cytokine, time of saliva collection, outcome, irradiation parameters, number of irradiation points, and timing of PBM. These data were then arranged into tables.

## 3. Results

### 3.1. Screening Results

**Figure 1:**
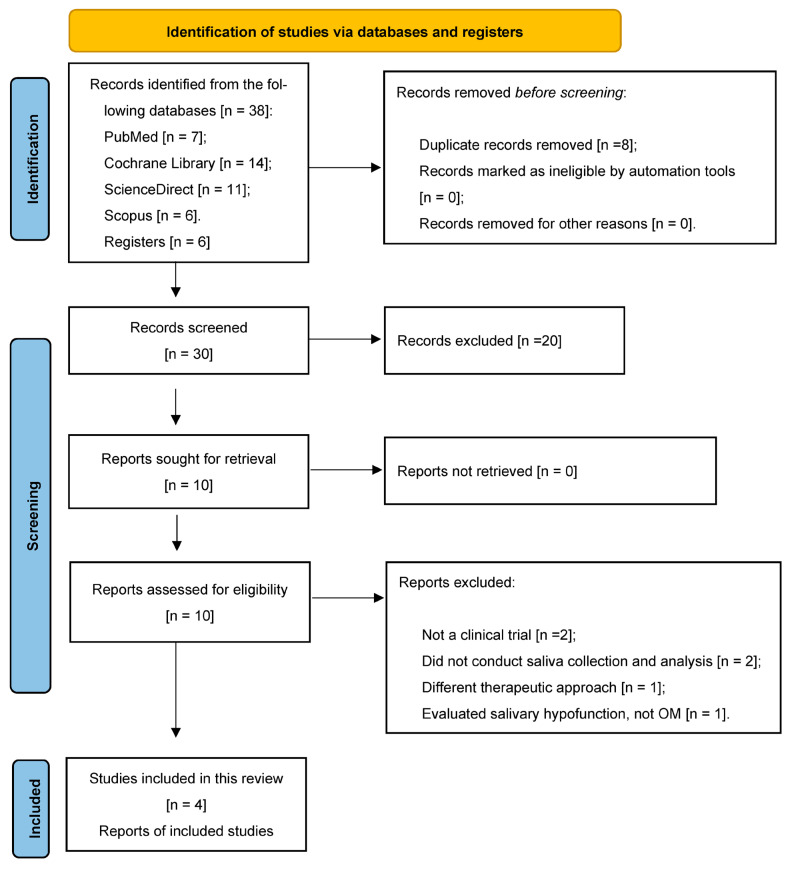
PRISMA flowchart for the included studies.

Records were identified from 38 studies [7 PubMed, 14 Cochrane Library, 11 ScienceDirect, and 6 Scopus]. Only four articles fit the selection criteria and were included for a qualitative analysis and data extraction. This review excluded eight parallel (duplicate), twenty-three inconsistent, one collaborative, and six studies based on the preprint criteria, as shown in [Figure 1]. Furthermore, 76 papers were used as background and discussion material.

All the studies included in this review were clinical trials conducted in Brazil [19,20,21,22]. The type of tumor was head and neck cancer in the studies of both Oton-Leite et al., 2015 [20], and Martins et al., 2021 [22]. In the study by Silva et al., 2015 [19], and Salvador et al., 2017 [21], hematological malignancies were found. Regarding the anticancer treatment, it was radiotherapy with or without chemotherapy, according to Martins et al., 2021 [22]. Patients received chemoradiotherapy in the study by Oton-Leite et al., 2015 [20]; stem cell transplantation was performed in the study by Silva et al., 2015 [19], and Salvador et al., 2017 [21]. The trial comparison groups slightly differed between the reviewed trials. The groups included no treatment, sham radiation, only oral hygiene, and a placebo with a preventative oral care program. The number of patients ranged from 25 to 51. All the studies included in this review used an InGaAlP diode laser with a wavelength of 660 nm. Oton-Leite et al., 2015 [20], and Martins et al., 2021 [22], used the same irradiation parameters, irradiation points, and timing. The power was 25 mW, the energy density was 6.2 J/cm^2^, and the total number of irradiation points was 61 in the mouth for 10 s for each point. Silva et al., 2015 [19], and Salvador et al., 2017 [21], used the same irradiation parameters, irradiation points, and timing. The power was 40 mW, the energy density was 4 J/cm^2^, and the total number of irradiation points was 10 points distributed in the mouth for 4 s for each point [Table 1].

### 3.2. Outcome

All the studies used the World Health Organization (WHO) mucositis scale. Oton-Leite et al., 2015 [20], and Martins et al., 2021 [22], used the National Cancer Institute scales in addition to the previous scale. All the studies showed a reduction in the severity of mucositis seen in the PBM group. Regarding the type of cytokines studied, Oton-Leite et al., 2015 [20], and Silva et al., 2015 [19], studied the same cytokines, while Martins et al., 2021 [22], studied IL-6, IL-8, IL-10, IL-12p70, IL-1β, and TNF-α. Salvador et al., 2017 [21], only studied one cytokine type, IL-8. Oton-Leite et al., 2015 [20], and Silva et al., 2015 [19], used an enzyme-linked immunoassay test (ELISA). The other two authors used a cytometric bead array analysis. The results varied between the studies included in this review. Silva et al., 2015 [19], found no effect of PBM on salivary cytokines. However, Oton-Leite et al., 2015 [20], found that PBM decreased interleukin six levels. Salvador et al., 2017 [21], also saw an effect of PBM on salivary cytokines, but the only cytokine studied was interleukin 8. Martins et al., 2021 [22], reported that PBM therapy promoted increased concentrations of IL-12p70, TNF-α, and IL-10 [Table 2].

### 3.3. Risk of Bias Assessment

**Figure 2:**
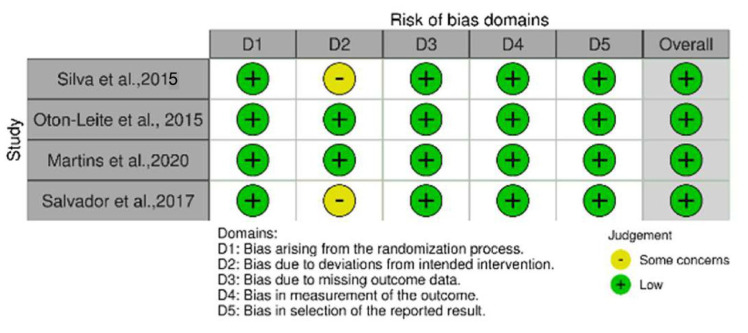
Cochrane Risk of Bias for Randomized Trials (RoB 2.0) tool. The table was generated using the website (https://www.riskofbias.info/welcome/robvis-visualization-tool, accessed on 5 March 2024) [19,20,21,22].

The risk of bias in the studies included in this review was assessed using the Revised Cochrane Risk of Bias for Randomized Trials (RoB 2.0) tool [Figure 2]. Each study was individually evaluated using QUADAS-2 to determine the risk of bias and applicability concerns [Table 3], and the Jadad scales for reporting randomized controlled trials to appraise methodological quality [Table 4]. The assessment included evaluating the risk of bias arising from the randomization process, the risk of bias due to deviations from the intended interventions, the risk of bias due to missing outcome data, the risk of bias in the measurement of the outcome, and the risk of bias in the selection of the reported results. 

## 4. Discussion

Oral mucositis is the most debilitating and bothersome side effect of non-surgical anticancer therapy. The mechanism of the occurrence of OM is complex, but in a simplified and brief way, it is associated with an elevated level of local reactive oxygen species (ROS) [23,24,25]. ROS provide essential protective tools for health conditions, including their involvement in the phagocyte-mediated killing of microorganisms [26,27].

However, when the balance within the generated ROS is disturbed, key transcription factors, such as activation of transcription 3 (STAT3), nuclear factor B (NFкB), and signal transducer, in turn stimulate the production of tumor necrosis factor (TNF), interleukin 1 (IL-1), and interleukin 6 [IL-6] pro-inflammatory cytokines, which ultimately leads to OM [23,24,25]. These mediators create a chain reaction that causes even more tissue damage. During its amplification phase, OM may have an inflammatory infiltration of the macrophages, neutrophils, and mast cells. Furthermore, bacterial wound colonization is prevalent and may produce additional inflammatory cytokines, leading to the most severe state of OM [24,25].

Cytokine levels in the biological fluids of patients with OM have been debated in several studies. Indeed, cytokine levels in biological fluids and OM development have demonstrated a significant relationship. Among the cytokines that lead to the development of OM, IL-1β and IL-6 have been highlighted as essential factors in the process [28,29]. Increasing radiation doses have resulted in elevated concentrations of TNF-a and IL-6 [30], and reduced salivary EGF levels [31,32,33,34,35] in patients with OM receiving RT. Additionally, a favorable correlation of IL-6 levels with OM severity has been observed in patients receiving combination chemotherapy [36]. Min et al. also showed that complications in patients during HSCT correlate with an elevated level of IL-6 in the blood [37]. 

Ye et al. [38] found that IL-8 is associated with an increased risk of OM in CT patients. In the same context, Citrin et al. [30] argued that OM in patients receiving chemoradiation for head and neck malignancies is related to reduced salivary IL-10 levels. 

According to this systematic review, Silva et al. and Oton-Leite et al. found that IL-6 is the most relevant inflammatory mediator for the development and severity of OM. Among the studied cytokines, Martins et al. found that higher OM scores were associated with higher levels of IL1-β and lower levels of TNF-α, IL12p70, and IL-10. Salvador et al. found that severe OM was related to the elevation of the only cytokine they studied, IL-8.

PBM is a safe treatment option. No undesirable effects have been observed on overall or disease-free survival, and local disease recurrence has been observed [39,40,41,42]. In contrast, [42] it has been indicated that PBM may increase the survival of patients with head and neck cancer, possibly due to a reduced number of interruptions to the cancer treatment [43,44]. Many studies have shown PBM’s positive role in preventing and treating OM [14,45,46]. Due to its positive effects, it has been recommended for use in patients undergoing non-surgical anticancer treatment by both WALT and MASCC/ISOO.

Although two different irradiation protocols were used in the studies included in this review, PBM showed positive results in reducing the severity of OM in all the studies. Despite utilizing different irradiation protocols, they all fell within the recommended limits. The WALT guidelines recommend using an LED/laser device with a visible wavelength of 630–680 nm to prevent mucositis [10,45]. Also, the PBM output of 10–100 MW power was within the range recommended by Bensadon et al. in their meta-analysis [47]. Also, the frequency of application was among what was recommended by Cronshaw et al., who recommended applying PBM at least twice a week [10].

Despite the emphasis on the role of PBM in managing OM, the precise mechanism by which it works still needs to be determined. Experimental studies have showed that PBM can alter the response to tissue repair in both in vitro and in vivo conditions [48,49,50,51,52,53,54] and pro-inflammatory and anti-inflammatory cytokine levels [55,56,57,58,59,60]. It has been observed that PBM can reduce inflammatory cell migration [48,59], as well as TNF-α [55,56,57,59], COX-2 [58], IL-1β [53,56,58,59,60], and IL-6 [56,57,58,59,60] cytokine levels, which have been shown to contribute to a general decrease in the inflammatory response in animal models of many conditions, including tendonitis [48,58], osteoarthritis [59], and acute inflammation [55,56,60].

However, studies evaluating the effect of PBM on cytokines in humans have been limited. Reviewing the literature revealed that only four studies assessed the impact of PBM on cytokines in the saliva of patients with OM, and all had a low risk of bias.

Studies that used saliva samples were selected for this review due to the advantages of saliva in terms of the ease of collection compared to plasma and other bodily fluids [61,62]. In addition, cytokine levels in saliva more accurately manifest the immune environment’s local activity in the oral mucosal compared to its systemic activity [61].

### 4.1. IL-6

IL-6 has a variety of biological activities. It acts as both an anti-inflammatory and pro-inflammatory cytokine. It induces the degradation of tissue via matrix metalloproteinase activation. Furthermore, IL-6 has a role in increasing vascular permeability and the migration of inflammatory cells, like macrophages [57,58]. 

Furthermore, IL6 is secreted from many cell types in a wound environment. It increases fibroblast proliferation at the site of injury, and it has been shown to have local and systemic effects on wound healing [63].

Oton-Leite et al. concluded that the lesser the IL-6 concentrations, the lower the mucosal damage. Arguing that IL-6 inflammatory mediators have a crucial role in the severity of OM, they noted in their trials that the PBM study group experienced lower concentrations of IL-6 compared to the control group. However, Martins et al. and Silva et al. found no significant differences in saliva IL-6 levels between the control and laser groups.

### 4.2. IL-10

The anti-inflammatory cytokine IL-10 is produced by T lymphocytes, which inhibit the production of pro-inflammatory cytokines and prevent neutrophils and macrophages from infiltrating an infection [37,63,64,65]. According to this systematic review, Silva et al. and Oton-Leite et al. found no significant differences in salivary IL-10 levels between the control and laser groups. However, Martins et al. suggested the role of PBM in increasing salivary IL-10, attributing this to the role of PBM balancing pro- and anti-inflammatory cytokines, enabling a more effective healing process.

### 4.3. IL-8

Interleukin 8 is a cytokine released by different cell types in our body, like lymphocytes, neutrophils, macrophages, fibroblasts, keratinocytes, monocytes, epithelial cells, and endothelial cells [66,67,68,69]. IL-8 has a chemotactic effect on macrophages and monocyte-derived neutrophils, promotes epithelial cell proliferation and migration, and stimulates the expression of metalloproteinases in leukocytes [70].

Salvador et al. observed lower levels of this cytokine in patients treated with PBM. It has been hypothesized that a decrease in IL-8 could reduce the migration of neutrophils, macrophages, and other inflammatory cells, and the production of enzymes, cytokines, and reactive oxygen species in inflamed oral mucosa, thus reducing tissue damage and achieving the clinical improvement of OM [67,68,71]. However, Martins et al. found no effect of PBM on salivary IL-8.

### 4.4. IL-1β

IL-1β is one of the pro-inflammatory cytokines. It is produced by monocytes, dendritic cells, and macrophages, and can stimulate a Th1 immune response and produce IL-6 [72]. Despite the significance role of this cytokine in immune response, none of the studies included in this review indicated any significant change in the levels of this cytokine due to PBM. 

### 4.5. TNF-α

TNF-α [tumor necrosis factor-alpha] is a cytokine with pleiotropic effects on various cell types. TNF-α was first identified as a factor that promotes tumor necrosis, but it has lately been discovered to have additional significant activities. It is an essential regulator of inflammatory responses and has been linked to the development of several inflammatory and autoimmune illnesses [73]. TNF-alpha induces inflammation by activating pro-inflammatory responses in capillary endothelial cells, allowing leukocyte adhesion and infiltration [74].

In Martins et al.’s study, higher levels of this cytokine were found in the group that received PBM. Thus, illustrating the role of PBM in activating inflammatory cells to balance the inflammatory response. However, Silva et al. and Oton-Leite et al. found no significant differences in this cytokine’s levels in the control and laser groups.

### 4.6. IL-12p70

IL-12p70 is a cytokine secreted by dendritic cells and macrophages associated with the cytotoxic immune response [75]. Martins et al. observed that the concentration of IL-12p70 in saliva was higher in the PBM group. The expression of toll-like receptors (TLRs) has been reported to be associated with reduced mucositis rates [76,77]. 

IL-12p70 is probably induced by TLR+ antigen-presenting cells stimulated by PBM, thus reducing the mucosal aggressive agents, and enhancing the antimicrobial response.

### 4.7. TGF-β

Transforming growth factor-beta [TGF-β] has several primary functions: it can induce the growth of mesenchymal cells, extracellular matrix formation, inhibit other cellular functions, and resolve inflammatory reactions due to its chemoattraction for inflammatory cells and fibroblasts, which relate to its role in wound healing [78]. Oton-Leite et al. found a slight reduction in anti-inflammatory cytokines (TGF-β) in the PBM group compared with the control group at almost all evaluated times. Pires et al. [58] suggested that the reduction in TGF-β induced by the laser treatment may be an indirect response to a decreased level of pro-inflammatory cytokines. 

The results regarding cytokine levels were different among the studies included in this review, likely due to the considerable variation between these studies in several aspects. It starts with the type of anticancer treatment. The difference in treatment patterns could be the most prominent reason for the differences in cytokine levels between the studies. Salvador et al. argued that the biological effect on OM differs between patients undergoing radiotherapy alone and chemotherapy patients undergoing HSCT. HSCT compromises cytokine production and inflammatory responses, like the activation and migration of neutrophils.

Even though the number of selected clinical trials was limited, there are notable discrepancies in the studies’ results, mainly due to differences in the analysis methods, such as cytometric bead array analysis and the ELISA test [79]. Furthermore, variations in saliva sampling times between the studies made it challenging to make quantitative comparisons. It is worth mentioning that the selected trials exhibited some risk of bias.

## 5. Conclusions

Our results show that photobiomodulation has demonstrated positive results for reducing the severity of OM in the included studies. Among the examined salivary cytokines, IL-6 is the most relevant cytokine for oral mucositis development and severity. A variation in the cytokine levels between the studies was noted, due to differences in the type of anticancer treatment and saliva sampling. Hence, further studies are needed. 

## Figures and Tables

**Table 1:** General characteristics of the included studies with detailed information about the subjects and intervention methods.

Author/Date	Tumor\ Therapy	Sample Size	Comparison Group	Type of PBM Device	Wavelength	Energy Density	PBM Duration	Power	Irradiation Points	Timing
Silva et al., 2015 [19]	Hematologic malignancies \High-dose chemotherapy, with or without radiation therapy.	30 patients	None	InGaAIP	660 nm	4 J/cm^2^	4.0 s per point	40 mW	“The tip touched the mucosa of the lips, right and left buccal mucosa, right and left lateral tongue, ventral tongue, and buccal floor, giving 10 points per region.”	“Subjects received the PBM from the first day of the conditioning regimen and continued every day, until D+7.”
Oton-Leite et al., 2015 [20]	Head and neck cancer\ Chemoradiotherapy.	25 patients	Placebo	InGaAlP diode laser	660 nm	6.2 J/cm^2^	10 s at each point	25 mW	“Buccal mucosa [10 points on each side], lips [8 points on upper and lower internal mucosa], hard palate [3 points], lateral tongue [10 points on each side], dorsal tongue [3 points], soft palate [3 points], floor of the mouth [2 points], and in the labial commissure [one point on each side].”	“The first session was performed on the first day of RT, and the following sessions occurred three times a week on alternate days, always before each session of RT, until the end of the treatment.”
Salvador et al., 2017 [21]	Hematologic malignancies \High-dose chemotherapy, with or without radiation therapy.	51 patients	Only the oral hygiene guidelines	InGaAlP	660 nm	4 J/cm^2^	4.0 s per point	40 mW	“A total of 10 points were spread over the upper and lower labial mucosa, right and left buccal mucosa, right and left lateral tongue, ventral of the tongue, and floor of the mouth.”	“Subjects received the PBM from the first day of the conditioning regimen and continued every day until the seventh post-transplant day [D+7].”
Martins et al., 2021 [22]	Head and neck cancer\ Radiotherapy, associated or not with chemotherapy.	48 patients	Placebo and preventative oral care program	InGaAlP diode laser	660 nm	6.2 J/cm^2^	10 s at each point	25 mW	“Right and left buccal mucosa [10 points on each side], upper and lower labial mucosa [4 points each lip], hard palate [3 points], the lateral surface of the tongue [10 points on each side], soft palate [3 points], dorsal tongue [3 points], floor of the mouth [2 points], and labia commissure [one point on each side]”	“The first session was performed on the first day of RT, and the following sessions occurred three times a week on alternate days, always before each session of RT until the end of the treatment.”

PBM: photobiomodulation; RT: radiotherapy.

**Table 2:** Cytokine evaluation and outcomes of the studies.

Author	OM Evaluation Method	Type of Cytokine	Time of Saliva Collection	Outcome
Silva et al., 2015 [19]	World Health Organization [WHO] mucositis scale	“TNF-α, IL-6, IL-1β, IL-10, TGF-β concentrations were assessed using ELISA test.”	“Samples were collected from patients on admission [AD], D-1, D+3, D+7, and on marrow engraftment day [ME].”	The OM lesions were clinically less severe in the PMB group [*p* < 0.05]. However, neither blood nor salivary inflammatory mediators demonstrated any statistically significant differences.
Oton-Leite et al., 2015 [20]	World Health Organization [WHO] and National Cancer Institute [NCI] scales	“TNF-a, IL-6, IL-1β, IL-10, TGF-β concentrations were assessed using the ELISA test.”	“Saliva samples were collected on admission, and on the 7th, 21st and 35th sessions of radiotherapy.”	Although no statistical significance can be drawn from the TGF-β, IL-10, IL-1β, and TNF-a concentrations between the PMB and control groups, a reduction trend was observed. The PMB group experienced a significant reduction in IL-6 concentration, especially following the 35th session.
Salvador et al., 2017 [21]	World Health Organization [WHO] mucositis scale	“CXCL8/interleukin 8, using cytometric bead array analysis.”	“Saliva samples were collected at the time of admission [AD], on the 7th day after transplantation [D+7], and on the day of discharge [with neutrophil > 0.5 × 10^9^/L for two consecutive days] [HD].”	The CXCL8 chemokine in the PBM group was reduced by 85% at the 7th session, whereas there was an up to 70.8% increase in CXCL8 observed in the control group [*p* = 0.007]. From the 7th to the 11th sessions, MO severity was significantly reduced by PBM [*p* < 0.05].
Martins et al., 2021 [22]	World Health Organization and the National Cancer Institute scales	“IL-6, IL-8, IL-10, IL-12p70, IL-1β, and TNF-α were measured using the cytometric bead array.”	“Samples were collected in the 1st [baseline], 7th, 14th, 21st and 30th sessions of RT.”	PBM increased the concentration of IL-10, TNF-α, and IL-12p70. However, OM severity was noticeably reduced in the PBM group compared with the control group.

**Table 3:** Tabular presentation of QUADAS-2 results for evaluating risks of bias and concerns of applicability.

Study	Risk of Bias				Domains	Applicability Concerns			Domains
	Patient Selection	Index Test	Reference Standard	Flow and Timing		Patient Selection	Index Test	Reference Standard	
Martins et al., 2021 [22]	Low	Unclear	Unclear	Low	At risk of bias	Low	Low	Low	Low concerns regarding applicability
Salvador et al., 2017 [21]	Unclear	Unclear	Low	Unclear	At risk of bias	Low	Low	Low	Low concerns regarding applicability
Silva et al., 2015 [19]	Unclear	Unclear	Low	Unclear	At risk of bias	Low	Low	Low	Low concerns regarding applicability
Oton-Leite et al., 2015 [20]	Unclear	Low	Low	Low	Low risk of bias	Low	Low	Low	Low concerns regarding applicability

**Table 4:** The Jadad scale for reporting randomized controlled trials was used to evaluate the methodological quality.

Study	Dimension				
	Randomization	Blinding	An Account of All Patients	Total Score	
Martins et al., 2021 [22]	2/2	2/2	1/1	5/5	High quality
Salvador et al., 2017 [21]	2/2	1/2	1/1	4/5	High quality
Silva et al., 2015 [19]	1/2	1/2	1/1	3/5	Good quality
Oton-Leite et al., 2015 [20]	1/2	2/2	1/1	4/5	High quality

## Data Availability

The data supporting the conclusions of this article will be made available by the authors on request.
